# Frequency of Acute Kidney Injury (AKI) among patients presenting with Organophosphate Poisoning at National Poison Control Centre, Karachi: A prospective cross-sectional survey

**DOI:** 10.12669/pjms.38.3.4819

**Published:** 2022

**Authors:** Sadaf Hanif, Rukhsana Abdul Sattar

**Affiliations:** 1 Dr. Sadaf Hanif, FCPS. Department of Medicine, Ward – 5, Jinnah Postgarduate Medical Centre, Karachi, Pakistan; 2 Dr. Rukhsana Abdul Sattar, FCPS. Department of Medicine, Ward – 5, Jinnah Postgarduate Medical Centre, Karachi, Pakistan

**Keywords:** Organophosphate poisoning, Acute Kidney Injury

## Abstract

**Background and Objective:**

Organophosphates poisoning is among the most prevalent forms of intentional and unintentional poisoning in Pakistan. However, the actual burden of AKI secondary to organophosphate poisoning in Pakistani population is still not known. This study aimed to determine the actual burden of AKI among patients admitted at National Poison Control Centre, Karachi.

**Methods:**

A cross-sectional survey was conducted at National Poison Control Centre, Karachi November, 2013 to April, 2014. A sample of 300 patients of age 18 years and above, presenting with organophosphate poisoning within 24 of exposure or ingestion were included in the study. Frequency of acute kidney injury was calculated using the diagnostic criteria of serum creatinine level of >1.4 mg/dL. Data was analyzed using SPSS version 19.

**Results:**

The frequency of AKI which was defined as creatinine level >1.4 mg/dL was 22.3% (n=67). However, there was no statistically significant difference was found in frequency of AKI on the basis of age, sex, amount of organophosphates ingested and BMI. This study found statistically significant differences in the AKI frequency on the basis of lag time. Those who presented earlier after poisoning had relatively low frequency of AKI.

**Conclusion:**

This study concludes that AKI is a common complication among patients presenting with organophosphate poisoning at National Poison Control Center, Karachi. Lag time is a key determinant of AKI among patients with organophosphate poisoning. Timely treatment can prevent this critical complication among patients with organophosphate poisoning.

## INTRODUCTION

Organophosphates anti-esterase compounds widely used as insecticides in agricultural and domestic settings throughout the world.^1^ Organophosphate’s poisoning is a global public health problem due to easy access, unregulated sale leading to frequent use as an agent for deliberate self-harm.^1^,^2^ Organophosphate poisoning can be occurred through multiple routes of exposure including; ingestion, inhalation or transdermal absorption of the compound.

Organophosphates exposure causes severe clinical symptoms and may result in fatal clinical complications including respiratory failure, renal failure and cardiovascular collapse.^1^,^2^ Organophosphates exert their acute effects by causing overstimulation at cholinergic nerve terminals.^3^ The excess acetylcholine causes constant acetylcholine receptor triggering, resulting in malfunction of the autonomic, somatic and central nervous systems.^3^ OP poisoning lead to acute cholinergic crisis causing diarrhea, urinary incontinence, miosis, bradycardia, bronchorrhoea, bronchoconstriction, salivation, lacrimation, emesis, hypotension, cardiac arrhythmias, fasciculations, tremors, muscle weakness with respiratory failure, hypertension, tachycardia, sweating, mydriasis, altered level of consciousness and seizures.^3^,^4^ It also causes dysfunction of kidneys and may result in alarmingly raised serum creatinine levels indicating, Acute Kidney Injury (AKI) which is among the most frequent systematic complication following organophosphate poisoning.^4^,^5^ Hence, intoxicated patients should receive immediate support, adequate hydration and immediate correction of acid-base balance to reduce the risk of AKI following organophosphate poisoning.^5^,^6^

Pakistan is among the countries bearing considerable burden of organophosphate poisoning due to poor control over unregulated access and insufficient training and knowledge to handle pesticides in occupational settings.^7^-^9^ Organophosphates poisoning is among the most prevalent forms of intentional and unintentional poisoning resulting in morbidity and mortality.^8^-^10^ However, the actual burden of AKI secondary to organophosphate poisoning in Pakistani population is still not known. This study will determine the frequency of AKI among patients presenting with organophosphate poisoning at National Poisson Control Center in Karachi. Determining the actual magnitude or burden of AKI among organophosphate poisoning patients will help in identifying and developing context specific treatment guidelines.

## METHODS

A cross-sectional study was conducted between November, 2013 to April, 2014 at National Poisoning Centre, Medical Unit-I; Jinnah Postgraduate Medical Center (JPMC), Karachi. A sample of 214 was calculated using Open Epi sample size calculator. Sample was calculated for a confidence interval of 95%, precision of 5% and an anticipated population proportion of 16.7% for AKI following organophosphate poisoning.^11^ Considering the possibility of high non-response rates due to emotional and mental instability of the patients or attendants we inflated the sample size to 300.

Patients of either gender admitted patients with organophosphate poisoning of age 18 years or above, admitted within 24 hours of organophosphate poisoning were included in the study using non-probability consecutive sampling. However, patients who were unconscious and having no attendant, previous medical history of cardiac disease, diabetes mellitus, hypertension, chronic renal injury, ketoacidosis due to diabetes mellitus and patients who were partially treated at some other facility and then referred, were excluded from this study. Body Mass Index (BMI) was also calculated using classification of World Health Organization.^12^ Furthermore, patients whose diagnosis was not confirmed due to suspicion of conditions which mimic organophosphate poisoning like, nicotine poisoning, botulin poisoning, and fungicide overdose poisoning were also excluded.

Data was collected from eligible study participants after taking informed consent. In case where the eligible participants had altered mental status or lack of consciousness, informed consent was obtained from patients` attendant available at the time of data collection. A structured questionnaires was used to collect the information regarding socio-demographic characteristics, duration of poisoning and lag time. For the sake of this study “Lag time” was defined as the time duration between poisoning and initiation of medical care. Clinical data regarding serum creatinine levels at 12 hours interval after the initial renal function assessment at the time of admission was obtained from patients` clinical file or medical record. AKI was diagnosed using Rifle criteria for diagnosing AKI. Hence, any organophosphate poisoning patient showing three times increases in serum creatinine or 75% reduction in GFR or if baseline serum creatinine is ≥1.4 mg/dL) the patient was diagnosed to have AKI.^13^

Data were analyzed using SPSS version 19. Continuous variables like age, lag time, amount of poison ingested and serum creatinine level were analyzed as mean ± Standard deviation. Frequencies & percentages were calculated for sex, BMI, AKI, duration of organophosphate poisoning or exposure. Chi-square test was applied to assess any significant differences in the frequency of AKI on the basis of differences in age group, sex and lag time. P-value of less than 0.05 was considered statistically significant.

### Ethical Consideration

This study was conducted after obtaining approval from Ethics Review Committee, JPMC with ERC Number F.2-81/2014-GENL/23717/JPMC. A letter of permission was also obtained from authorities from National Poisoning Centre, Medical Unit-I to collect the required information from patients.

## RESULTS

A sample of 300 hundred organophosphate poisoning patients was taken to assess the frequency of AKI in these patients after OP poisoning. Among all the study participants 54.67% (n= 164) were male while 45.33% (n=136) were females. The median age of study participants was 27.34 years (IQR; 8.3 years). Age categorization showed that 60.33% (n= 181) of the study participants were very young and aged between 18-28 years, 26% (n=78) patients were of age 29-38 years, while 13.7% (n= 41) patients were of age 39-48 years. BMI was calculated for each study participant and interpreted using WHO criteria for Asian population.^11^

This study found that 3.33% (n=10) of study participants were underweight, while a considerable proportion had BMI in normal range i.e. 41.3% (n=124). However, 38.3% (n=115) of study participants were overweight and 17% (n=51) were obese.

The study computed the mean amount of OP ingested by study participants which was approximately 77.51 milliliters with an SD of ± 43.24 milliliters. This study also calculated lag time which reflected approximate time duration between OP exposure or poisoning and reaching the emergency department. The study found mean lag time of 9.67 hours ± 6.02 hours. However, 78% (n= 234) of the study participants reported a lag time of 12 hours; while 21% (n= 63) reported a lag time between 12 to 23 hours. Only 1% (n=3) patients had lag time between 23hours to 24 hours.

**Fig.1:**
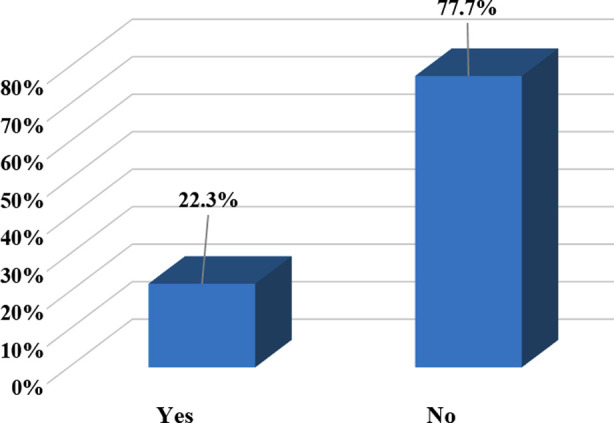
Frequency of Acute Kidney Injury among Organophosphate Poisoning Patients admitted at National Poison Control Center, Karachi (n=300).

The principal outcome of this study was frequency of AKI which was defined as creatinine level >1.4 mg/dL. Among all the study participants who were exposed to organophosphates 22.3% (n=67) developed acute kidney injury. (Fig.1) However, the means serum creatinine level of the study participants was 0.96 ± 0.58 mg/dl.

Age stratified analysis was done by categorizing study participants into three age groups i.e. 18 to 28 years, 29 to 38 years and participants with age of 39 years or above. Moreover, it was found that acute kidney injury was relatively more frequent in the youngest age group i.e. 18 to 28 years and then age group between 29 to 38 years with a frequency of 25.4% and 20.5% respectively. The frequency of AKI among participants of age 39 years and above was 12.2%). However, the apparent difference in the frequency of AKI among different age groups was not statistically significant (p-value; 0.168) (Table-I).

**Table-I:** Frequency distribution of Acute Kidney Injury following organophosphate poisoning as per different characteristics, among patients admitted at National Poison Control Center, Karachi (n=300).

	Yes	No	p-value
***Age***			
18-29 years	46 (25.6)	135(74.3)	0.168
30-38 years	16(20.5)	62 (79.5)	
39 years and above	05 (12.2)	36(87.8)	
***Gender***			
Male	39(23.7)	125(76.3)	0.302
Female	28(20.6)	108(79.4)	
***Lag Time***			
Up to 12 hours	48(20.5)	186(79.5)	0.004*
More than 12 hours to 23 hours	14(22.2)	14(77.8)	
More than 23 hours to 24 hours	03(100)	0	

* p-value of less than 0.05 was considered statistically significant.

The study found that frequency of AKI among males was 23.7% which was relatively higher as compared to frequency of AKI among females 20.6%. However, these findings were statistically insignificant (p-value- 0.302).

The frequency of AKI among those with a lag time between 12 hours to 23 hours and lag time between 23 hours to 24 hours was reported as 22.2% and 100.0% respectively. Nevertheless, this study found that study participants with a lag time of less than 12 hours for organophosphate poisoning had relatively lower frequency of AKI (20.5%) as compared to those with lag time between12 hours to 23 hours and lag time between 23 hours to 24 hours. This finding was statistically significant (p-value; 0.004) (Table-I).

## DISCUSSION

This study is among the very few research studies focusing AKI following Organophosphate poisoning. However, to the best of our knowledge this study is among few studies specifically measuring the frequency of AKI secondary to organophosphate poisoning in Pakistan. This study found that 22.3% of all patients who presented with organophosphate poisoning at National Poison Control Center, developed AKI with serum creatinine levels of more than >1.4mg/dL. This finding provides sufficient evidence that a considerable proportion of organophosphate poisoning patients suffer from AKI as a complication which is in line with previous studies.^14^-^17^ However the frequency of AKI following organophosphate poisoning, reported in current study is slightly higher than previous studies which can be explained by the inherent differences in study populations and study methods.^14^,^16^,^18^

The more than 50% of organophosphate poisoning patients included in this study were males where majority of the participants were between 18 to 40 years of age. This finding supports the natural epidemiology of organophosphate poisoning among developing countries with which shows relatively higher incidence of organophosphate poisoning between ages of 18 to 40 years.^19^-^21^ Furthermore, higher vulnerability of young males to all kind of injuries including suicide and accidental or occupational poisoning explains the higher proportion of males in this study.^22^ Similarly, frequency of AKI was higher among males as compared to females. Nevertheless, these findings were not statistically significant and large scale studies are required to establish the role of sex in determining the risk of AKI following organophosphate poisoning. Similarly, this study didn’t find any significant differences in the frequency of AKI among patients with different BMI. This finding is in contrast to previous evidence supporting the role of BMI in prognosis or recovery of patients admitted with organophosphate poisoning.^23^

This study also calculated lag time for each study participant which refers to the time duration between poisoning and initiation of medical care. The stratified analysis showed a statistically significant higher in frequency of AKI among the participants with relatively prolonged lag time as compared to groups with relatively short lag time. This finding is well supported by the previous studies identifying lag time of more than 24 hours as a risk factor for complication and mortality following organophosphate poisoning.^11^,^22^-^25^ This can be explained by the possible inaccuracies in calculating approximate amount of organophosphate exposure as well differences in the routes of exposure to organophosphates. Large scale studies with advanced methodology are required to assess the possible role of amount and route of exposure in development of AKI following organophosphate poisoning. On the other hand, this study identified the burden of AKI among patients presenting with organophosphate poisoning which is the most common cause of admission at National Poison Control Center.^22^ This study also highlighted the role of timely medical care in determining frequency of renal complications following organophosphate poisoning. The National Poison Control Center is the biggest specialized center to control poisoning cases in Sindh. It mainly receives patients from Karachi and surrounding urban, peri-urban as well as rural population across the province. There is need to establish more of such facilities to reduce lag time for ultimate decrease in renal and other systematic complications and associated mortality among patients presenting with organophosphate poisoning.^24^

### Limitations of the study

First, it includes data from one specialized health facility serving a huge catchment population. The study must have missed the organophosphate poisoning cases who received treatment at some other facility resulting in underestimation of the actual burden of AKI and limiting generalizability of the findings. Moreover, this study didn’t collect information regarding co-morbids as well as pre-existing renal problems among the study participants which limits interpretation of findings. Nevertheless, this study didn’t collect information regarding many potential effect modifiers including exact mode or route of transmission and previous history or exposure to Organophosphates. Furthermore, creatinine level after 12 hours interval level was the only clinical parameter applied to identify acute renal injury. In last the possibility of information bias cannot be ruled out because for very sick patients information was collected from their attendants. This might have also affected the measurement for amount of organophosphate actually consumed by the patient. However, despite few limitations this study brings new insights related to organophosphate poisoning and related management challenges in the local context. Large scale analytical studies need to be conducted to identify the specific risk factors associated with AKI following organophosphate poisoning. This will help in developing and identifying targeted social and medical interventions.

## CONCLUSION

AKI is a common complication among patients admitted with organophosphate poisoning. Timely medical care or treatment can help in preventing AKI. Controlled access to organophosphates, promoting and providing occupational safety and relevant training as well improved access to quality medical care can reduce the burden of AKI associated with Organophosphate Poisoning.

### Authors’ Contribution:

**SH** data collection, statistical analysis, manuscript writing and is responsible for integrity of the study.

**RS** conceived the idea and reviewed the manuscript.
